# Dictionary of Computing

**Published:** 1986-08

**Authors:** J. D. Davies

**Affiliations:** Department of Pathology University of Bristol


**DICTIONARY OF COMPUTING**

**Eds. V. Illingworth, E. L. Glaser and I. C. Pyle**
**Oxford University Press, Oxford, 1985 (reprint of 1st Ed., 1983)**
**pp. 393+xi, hardback. ISBN 0–19–853905–3.**
**Price £17.50**

Computer buffs need read no further. They will already be well aware of the virtues of this book. This review is not written for the buff. It is an attempt to explain why ordinary mortals might think of dipping into another language. Or perhaps, one should say another way of life.

There are many variants of standard English. These have in the past been dismissively labelled as jargon, argot, or mere regional variations of the English language. The individual characteristics of these variants of standard English are now being taken more seriously than was the cases thirty years ago (*vide:* E. Partridge's *A Dictionary of Slang and Unconventional English*, Routledge & Kegan Paul, London, 5th Edn., 1961).

All living languages are continuously changing. It is often difficult to keep up with them (see Phillip Howard on English most weeks in *The Times*). The other night on the television the use of a pejorative phrase, implying that someone was in less than first-class physical condition, was expressed by the Anglo-American string of words: 'you are like a plate of out-of-shape couch potatoes'. Now this singular phrase obviously has impact. The younger members of the family were quick to point out that 'couch' in this context means the type of food eaten in front of the television set, apparently whilst sitting on a couch.

The Dictionary of Computing is equally extraordinary. That is not to say that the book is filled with terms of insult. It most certainly is not. It is an attempt to explain the words which are used in the new world of computers, word processors and in the information explosion with which we are all threatened in medicine, as has already been indicated in an earlier issue of this *Journal* (1985 100 pp. 92–111).

Let me give some indication of what lies ahead. You might innocently think that the word adder, appearing in the first few entries of the book was fair play. An apparently homely half-adder is also included. These two beings are not snakes, or even human arithmeticians. They are not that at all, being devices performing the 'modulo-two addition on two binary digits, ... and its implementation that has provision only for input of added bits and (yet) is capable of generating sum and carry outputs'.

Thus whetting your appetite for more, I am certain that you can bearly restrain yourselves to know, over 300 pages later, exactly what windowing (sometimes better known as scissoring) is. The complexities of wired logic thereafter will seem simple child's play.

Please do not let me put you off. If you are already playing with a home computer, or slaving on a word processor at work this book will have an irresistable appeal. If not, it might still provide hours of incomprehensible amusement. I can think of few better signs that the world is fast going to the dogs (or the do loops, see p. 115).

J. D. Davies
Department of Pathology
University of Bristol

